# Biocompatibility and Application of Carbon Fibers in Heart Valve Tissue Engineering

**DOI:** 10.3389/fcvm.2021.793898

**Published:** 2021-12-24

**Authors:** Yuan-Tsan Tseng, Nabil F. Grace, Heba Aguib, Padmini Sarathchandra, Ann McCormack, Ahmed Ebeid, Nairouz Shehata, Mohamed Nagy, Hussam El-Nashar, Magdi H. Yacoub, Adrian Chester, Najma Latif

**Affiliations:** ^1^Heart Science Centre, Magdi Yacoub Institute, Harefield, United Kingdom; ^2^Imperial College London, National Heart and Lung Institute, London, United Kingdom; ^3^Centre for Innovative Materials Research, Lawrence Technological University, Southfield, MI, United States; ^4^Biomedical Engineering and Innovation Laboratory, Aswan Heart Centre, Aswan, Egypt

**Keywords:** carbon fibers, biocompatibility, heart valve, tissue engineering, biomaterials, composite, adipose-derived stem cells

## Abstract

The success of tissue-engineered heart valves rely on a balance between polymer degradation, appropriate cell repopulation, and extracellular matrix (ECM) deposition, in order for the valves to continue their vital function. However, the process of remodeling is highly dynamic and species dependent. The carbon fibers have been well used in the construction industry for their high tensile strength and flexibility and, therefore, might be relevant to support tissue-engineered hearts valve during this transition in the mechanically demanding environment of the circulation. The aim of this study was to assess the suitability of the carbon fibers to be incorporated into tissue-engineered heart valves, with respect to optimizing their cellular interaction and mechanical flexibility during valve opening and closure. The morphology and surface oxidation of the carbon fibers were characterized by scanning electron microscopy (SEM). Their ability to interact with human adipose-derived stem cells (hADSCs) was assessed with respect to cell attachment and phenotypic changes. hADSCs attached and maintained their expression of stem cell markers with negligible differentiation to other lineages. Incorporation of the carbon fibers into a stand-alone tissue-engineered aortic root, comprised of jet-sprayed polycaprolactone aligned carbon fibers, had no negative effects on the opening and closure characteristics of the valve when simulated in a pulsatile bioreactor. In conclusion, the carbon fibers were found to be conducive to hADSC attachment and maintaining their phenotype. The carbon fibers were sufficiently flexible for full motion of valvular opening and closure. This study provides a proof-of-concept for the incorporation of the carbon fibers into tissue-engineered heart valves to continue their vital function during scaffold degradation.

## Introduction

Tissue-engineered heart valves offer the potential to overcome the limitations of current prosthetics. The success of tissue-engineered heart valves rely upon several factors: first, the scaffold material being strong and flexible enough to withstand the hemodynamic cycle of loading and unloading at a frequency corresponding to a range of heart rates during rest and exercise. Second, the construct needs to be receptive to the population by cells either seeded during *in vitro* production of the valve or following implantation. Last, the scaffold should be biodegradable to allow the replacement of the own extracellular matrix (ECM) of the hosts. However, the process is highly dynamic and species dependent (1). Rapid cellular ingrowth and ECM deposition are often observed in animal models, but these observations typically fail to be observed in humans (1–4). Therefore, the potential risk for scaffold degradation and fatigue to occur prior to sufficient laying down of functional ECM leads to structural failure of the constructs.

Textile support as part of heart valve leaflets has been proposed previously by our group and others (5–9). However, most of the literature has been focused on reinforcement of the leaflet with mono- or multifilament yarns, which will still suffer from creep, fatigue, and unpredictable degradation over time. To ensure the stability of the construct during the remodeling process, we have assessed the suitability of incorporating carbon fibers into tissue-engineered heart valves to reinforce the biodegradable scaffold. The carbon fiber is a thin fiber between 5 and 20 μm in diameter composed of mostly carbon atoms. It has been used in the repair of damaged tendons and ligaments to provide additional support and strength during surgical repair and regeneration (10–12). More recently, the carbon fibers have been used to provide additional strength in scaffold materials used for bone, cartilage, and trachea tissue-engineered constructs (13–17). The low density and high strength properties of the carbon fibers, which are also flexible and have complete elastic recovery after unloading, give them excellent fatigue resistance (18). This profile of mechanical properties makes them good candidates for inclusion in scaffolds for heart valve tissue engineering.

The carbon fibers are usually combined with other polymers to reinforce the strength to weight ratio of the composite. This often required surface enhancement on the chemically inert carbon fiber surface that improves its chemical bonding and adhesion between the carbon fibers and matrix. Plasma oxidization is a simple and residue-free surface activation technique for the carbon fibers; thus, the focus of this *in-vitro* study was to investigate the biocompatibility of the carbon fibers in their pristine and plasma oxidized forms with human adipose-derived stem cells (hADSCs) to determine how binding of cells to the carbon fibers can be maximized. We have assessed the flexibility of the carbon fibers with respect to the motion of tissue-engineered valve cusps in a pulse duplicator. We envisage these findings that will provide a rationale for further studies into the use of carbon fibers as a part of composite scaffold to provide strength and durability of the engineered tissue.

## Materials and Methods

### Carbon Fibers

The carbon fibers used in this study were produced by the treatment of a polyacrylonitrile (PAN) precursor, with pyrolysis, surface treatment, and sizing processes (Toray Carbon Fibers Europe, Paris, and France). The size and shape of the carbon fibers were analyzed with a scanning electron microscope (SEM) to assess the uniformity of size and shape. For experiments with cells, the carbon fibers were sterilized by incubating in 70% ethanol for 1 h followed by washing in sterile phosphate-buffered saline (PBS) three times prior to cell seeding.

### Plasma Oxidation

The carbon fibers were mounted on a 24-well CellCrown™ 24 (Scaffdex Oy, Finland) and treated with plasma oxidation at 0.16 mbar oxygen (Diener Electronic, Germany). The carbon fibers were exposed to 30 W for 10, 20, and 30 min, which were compared to 30 min of 90 W. All the following analysis and cell seeding were performed within 24 h of plasma oxidization treatment.

### Cell Culture

The hADSCs were purchased from Lonza (PT-5006; Lonza, Switzerland) and cultured in a culture medium comprising adipose-derived stem cell basal medium, 10% fetal calf serum (FCS), 1% L-glutamine, and 0.1% gentamicin–amphotericin B (ADSC Growth Medium BulletKit^TM^, PT-4505; Lonza, Switzerland). The cells were fed every 3 days and subcultured at 90% confluency.

### Cell Seeding

The hADSCs (3 × 10^5^ cells) were simultaneously cultured on the pristine and plasma oxidized (30 W) carbon fibers (fixed on CellCrown™ 24) for 3 weeks under rotatory seeding at 10 rpm with a rotator (Bibby Scientific, UK) as described previously (19). In addition, the hADSCs (5,000 cells) were seeded on coverslips and cultured for 3 weeks as a control. At the end of this period, the coverslips and the carbon fibers were washed twice in PBS and fixed in 4% paraformaldehyde for 10 min. The fixative solution was removed with three rinses of PBS. The carbon fibers were removed from the CellCrown™ 24. Cells on coverslips and the carbon fibers were permeabilized with Triton X-100 (0.5% v/v in PBS) for 3 min and washed two times in PBS-Tween (PBS-T) (0.1% v/v). Cells were blocked using 3% (w/v) bovine serum albumin (BSA) and incubated with primary antibodies [alpha smooth muscle actin (α-SMA) (Dako, US), vimentin (Dako, US), calponin (Dako, US), SM22 (Abcam, US), vinculin (Sigma-Aldrich, US), Ectodysplasin A (EDA)-fibronectin (Dinova, Germany), alkaline phosphatase (Sigma-Aldrich, US), CD44 (BD Pharmingen™, US), osteocalcin (Abcam, US), CD105 (Abcam, US), CD90 (Dinova, Germany), CD31 (Dako, US), SRY-Box transcription factor 9 (Sox9) (R&D Systems, US), and peroxisome proliferator-activated receptor gamma (PPARγ) (Abcam, US)] in BSA 1.5% w/v for 1 h. After thorough washing in PBS-T, the cells were incubated with secondary antibodies for 1 h, washed 3 times during 5 min in PBS-T, and incubated 10 min with 4,6-diamidino-2-phenylindole (DAPI) (Sigma-Aldrich, US). Cells were washed again twice in PBS-T and mounted on glass slides in the PermaFluor Aqueous Mounting Fluid (Beckman Coulter, Fullerton, California, USA). Observations were performed with an inverted confocal microscope (Zeiss, LSM 510 Meta Inverted).

### Scanning Electron Microscopy

The hADSCs grown on coverslips and the carbon fibers were fixed in 2.5% glutaraldehyde in 0.1 M sodium cacodylate buffer for at least 2 h followed by two buffer washes. Specimens were then postfixed with 1% osmium tetroxide in 0.1 M sodium cacodylate buffer for 1 h. After two buffer washes, specimens were dehydrated through ascending series of ethanol starting from 25 to 100%. Then, the specimens were chemically dried using hexamethylenedizilasine (HMDS), mounted on SEM stubs, and coated with gold/palladium. Images of the carbon fibers with and without cells were taken on JEOL JSM-6010LA analytical scanning microscope. hADSCs on coverslips and the carbon fibers were added to aluminum sample holders with carbon tape, air dried overnight, and coated with gold/palladium. An energy dispersive X-ray analyzer energy dispersive spectroscopy (EDS) (JEOL JED-2300 X-ray Microanalysis System) was used to investigate the surface structure of the carbon fibers.

### Proliferation Assay

After 3 weeks of cell seeding with the carbon fibers, proliferation assays were carried out with the CellTiter 96® Aqueous Non-radioactive Cell Proliferation Assay Kit (Promega G-5421, US) by adding 20 μl of (3-(4,5-dimethylthiazol-2-yl)-5-(3-carboxymethoxyphenyl)-2-(4-sulfophenyl)-2H-tetrazolium) (MTS)/phenazine methosulfate (PMS) solution with 100 μl of dulbecco's modified eagle medium (DMEM) on cells. Plates were incubated for 1 h at 37°C, 5% carbon dioxide (CO_2_), and the absorbance was read at 490 nm.

### Mechanical Testing

Samples of the carbon fibers between 3 and 5 mm in length [measured using a caliper (Mitutoyo, Japan)] and cross-sectional area measured by SEM (JEOL JSM-6010LA) were subjected to uniaxial tensile testing (TA Electroforce TestBench, Minnesota, USA) at a speed of 0.1 mm/s. For each condition, 4 repeated samples, cut longitudinally, were measured. The resulting stress strain curve was fitted with six-order polynomial trend line. The gradient of elastic modulus was taken from the steepest curve.

### Analysis of Cusp Movement/Hinge Mechanism

It is well known that the carbon fibers suffer from brittle snap when bend in 90° angles against the direction of the carbon fibers; therefore, motion analysis of a human heart valve was conducted. The Aswan Heart Science Center Ethics Committee approval and informed consent were obtained to use the CT images from a normal adult individual (female, aged 54 years). The hinge range of movement of the aortic valve was measured using CT images (Siemens Somatom definition flash dual source multislice CT machine with retrospective ECG gating, slice thickness 0.6 mm, pitch 0.18, and gantry rotation time 0.28 s). Three-dimensional (3D) segmentation was used to reconstruct cusp and sinus shape (Mimics Innovation Suite 21 research edition, Materialize NV, Leuven, Belgium). The segmented model was rotated to visualize the leaflet and sinus side perpendicular to the leaflet and sinus plane (side view).

Three nadir points of the three sinuses were determined and a plane representing the annular plane was created (**Figure 5C**). A cross-section through the middle of the cusp as a vertical plan (perpendicular to the annular plane) was marked to each sinus and the movement of the cusp and sinus wall to this vertical plan was tracked at five points of the cardiac cycle (0, 10, 20, 30, and 40%) covering the complete systolic phase.

To identify the movement of the hinge, the angles and radii of each cusp and sinus were measured. The radii (R) of the best fit circles are recorded and curvature is calculated by the relation k = 1/R. A plane (Pp) perpendicular to the annular plane (Pa) through each of the nadirs was created to measure the angle of the tangent of each cusp at the nadirs to Pp and, thus, track its movement.

### Bioreactor Testing

To demonstrate their utility and functionality in a tissue-engineered valve construct, the carbon fibers were sutured using a standard needle into the hinge region of the jet-sprayed nanofibrous polycaprolactone (PCL) scaffold (20). The carbon fibers were not incorporated into the jet-spraying process because they are not compatible with the spinning process. In addition, carbon fibers are only required at regions of high stress to alleviating the stress on the nanofiber scaffold. The nanofibrous scaffolds were first constructed into a 3D functional valve root using a preparatory process (patent pending) followed by suturing the carbon fibers along the hinge to the belly region and halfway up toward the coapting edge in a defined spatial manner (5 equally spaced markers were used as a guide in the center of this region). Each strand consists of 50 individual carbon fibers. The carbon fibers were tethered on the outside edge of the commissure and a running stitch was stitched following the marked parallel lines (**Figure 6**). We sutured the carbon fibers in the radial direction to demonstrate the worst-case scenario in the carbon fiber movement in the radial direction. Valve roots with and without the embedded carbon fibers were subjected to a hydrodynamic pulmonary profile as set in ISO 5840 (20 mm Hg mean pressure, 70 bpm, 5 L/min cardiac output, and 35% systolic duration) using the Aptus® Bioreactors (Aptus Bioreactors, USA). High speed camera (500 frames per second) (Sony, Japan) was used to capture the opening and closure of the valve over cardiac cycles. The relative geometric orifice area was calculated using in-house matrix laboratory (MATLAB) code based on the percentage of the observed opened area over the maximum observable viewing area.

### Statistical Analysis

Data were tested for normality using the Kolmorogov–Smirnov test and the Shapiro–Wilk test. A two-tailed *t*-test was used to test the means between the different groups using the GraphPad Prism software, US. *p* < 0.05 was considered as statistically significant.

## Results

### Scanning Electron Microscopy Demonstrates the Carbon Fibers of Uniform Diameter and Structure

**Figure 1:**
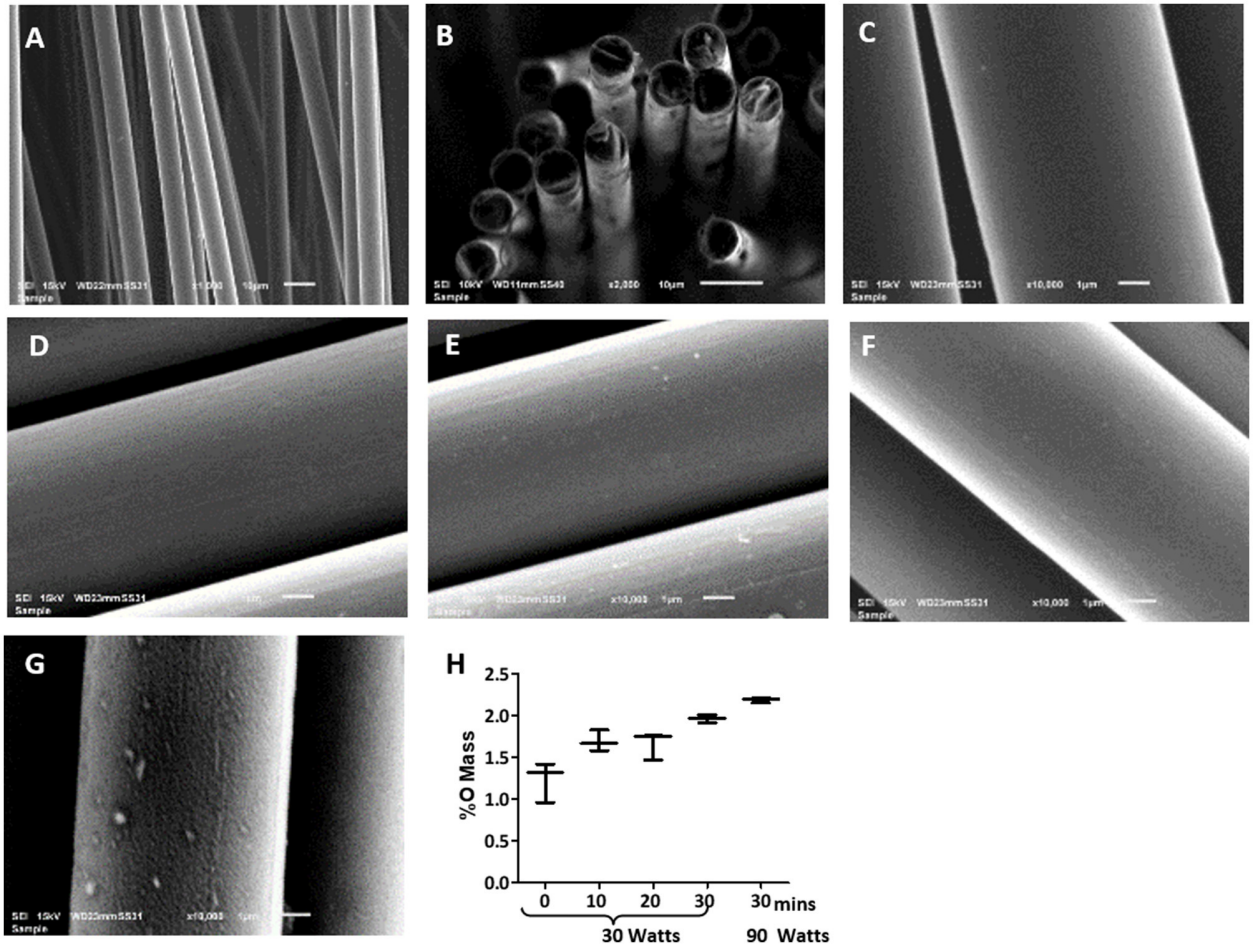
Scanning electron microscopy (SEM) images of uniform diameter and smooth surface of the carbon fibers. Images of the pristine carbon fibers as shown in **(A)** 1,000X, **(B)** 2,000X, and **(C)** 10,000X magnification show the pristine carbon fibers. Images of the carbon fibers with various oxygen plasma treatment, where **(D)** 10 min plasma oxidation at 30 W, **(E)** 20 min plasma oxidation at 30 W; **(F)** 30 min plasma oxidation at 30 W; **(G)** 30 min plasma oxidation at 90 W. Surface remains smooth up to 30 min of the oxygen plasma treatment at 30 W, but significant itching was observed with 90 W treatment. **(H)** shows the EDS analysis of the carbon fibers surface with the increase in oxygen content with increasing the time of plasma oxidation and the wattage.

The topology of the carbon fibers showed a uniform, smooth, and solid structure. It consisted of numerous individual carbon fibers (Figure 1A) with a uniform diameter of 7 μm. The carbon fibers had no visible defects such as cracks, pits, or splits, and no pores (Figure 1C). The cross-section of the carbon fibers showed a solid structure with no internal pores, although some staggering was observed due to uneven cutting and fracturing (Figure 1B).

### Plasma Oxidation Disrupts the Smooth Surface of the Carbon Fibers

Plasma oxidation modified the carbon fibers with an oxide surface layer. The EDS showed oxygen mass increased from 1.4 to 2% with increased treatment time from untreated to 30 min at 30 W (Figure 1H). This treatment maintained the smooth surface of the carbon fibers without any signs of damage (Figures 1C–F). However, an enhanced wattage to 90 W showed the surface becoming rough with random indentations (Figure 1G) and a marginal enhancement of oxide formation to 2.1%. Therefore, it is concluded that 30 min of 30 W plasma oxidation could be administered without any damage to the surface of the fibers. This level of plasma oxidation was used in subsequent cellular experiments with carbon fibers.

### Morphology of the hADSCs on Coverslips and the Carbon Fibers

**Figure 2:**
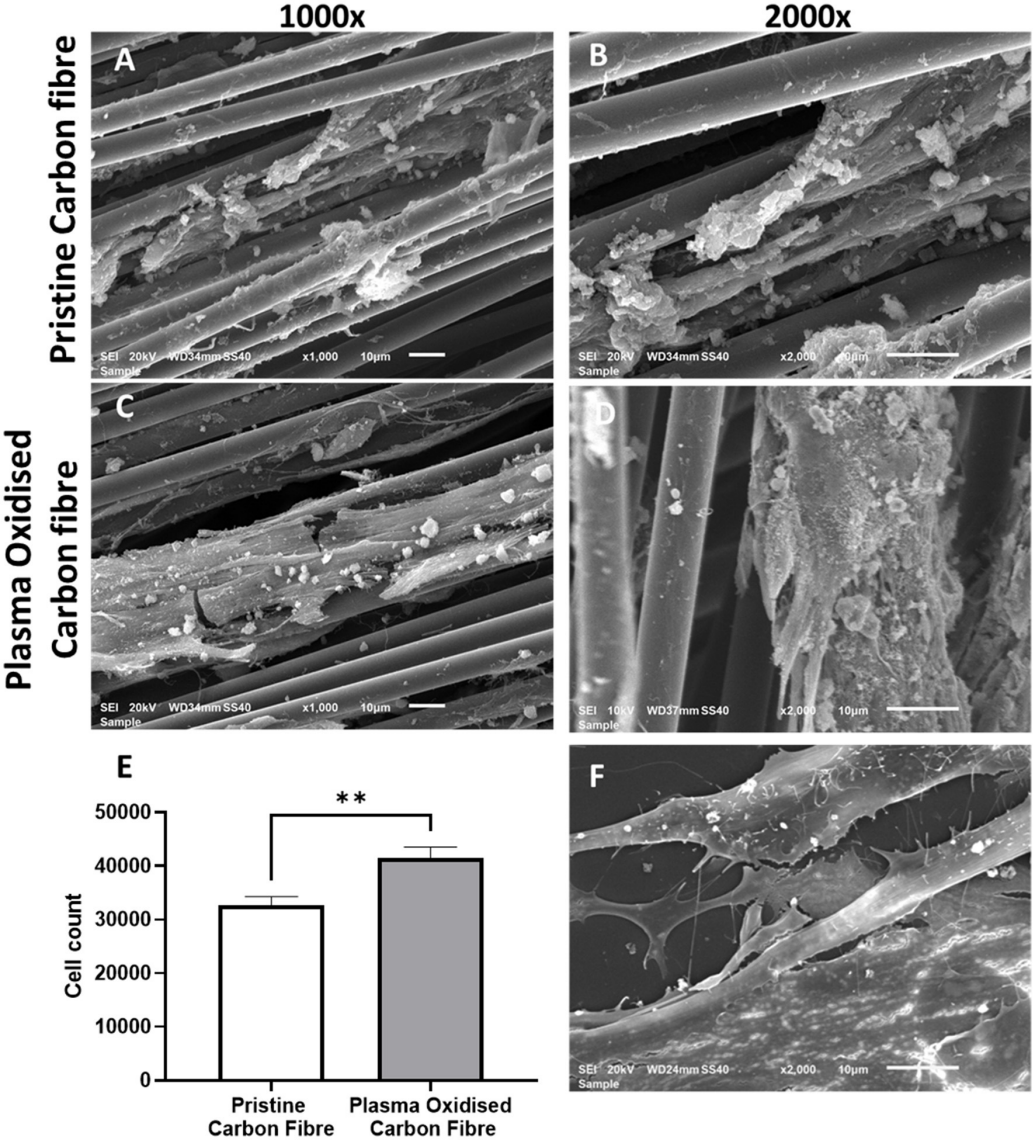
The SEM images of the cultured human adipose-derived stem cells (hADSCs) on the pristine carbon fiber at a magnification of 1,000X **(A)** and 2,000X **(B)**. **(C,D)** Show the cultured hADSCs on the plasma oxidized carbon fiber at a magnification of X1,000 and X2,000, respectively. **(E)** shows the proliferation (MTS) assay of the hADSCs on the pristine and plasma oxidized carbon fibers (^**^significant different with *p* < 0.05 base on the two-tailed *t*-test). **(F)** shows the SEM image of the control hADSCs on the coverslip at a magnification of X2,000.

The hADSCs were able to attach and spread on the carbon fibers in an aligned and elongated manner, along the length of both the pristine and plasma oxidized carbon fibers (Figures 2A–D). In addition, the hADSCs were able to wrap around a single carbon fiber as well as form a sheet of the hADSCs across the multiple fibers. Morphology of the hADSCs on the single carbon fibers was dissimilar to the hADSCs cultured on coverslips in such that they were elongated and spindly. The hADSCs grown on coverslips showed the typical flattened, spread out morphology with numerous filopodia extending from the cell surface. On reaching confluency, the hADSCs made good contact between adjacent cells with some overlapping of cells (Figure 2F).

### Cell Colonization to the Carbon Fibers

Cell colonization of the hADSCs on the pristine and plasma oxidized carbon fibers was performed with and without dynamic seeding. Static seeding of 3 × 10^5^ hADSCs to the carbon fibers resulted in poor adhesion, which was not quantifiable (not shown). This is most likely due to the settling of the hADSCs on the bottom of the well with little contact time to the carbon fibers. The dynamic seeding improved the contact time of cells to the carbon fibers resulting in quantifiable colonization. The MTS assay showed the cell colonization on the pristine carbon fibers (mean cell number 32,662, SD 1,609), which was further significantly improved by plasma oxidation (mean cell number 41,558, SD 1,982), *p* ≤ 0.05 (Figure 2E). The detected cell numbers in the non-plasma- and plasma-treated carbon fibers are 30 and 40 K, respectively. Therefore, the attachment efficiency is <10%, as there would be some proliferation.

### The Phenotype of the hADSCs on the Carbon Fibers

**Figure 3:**
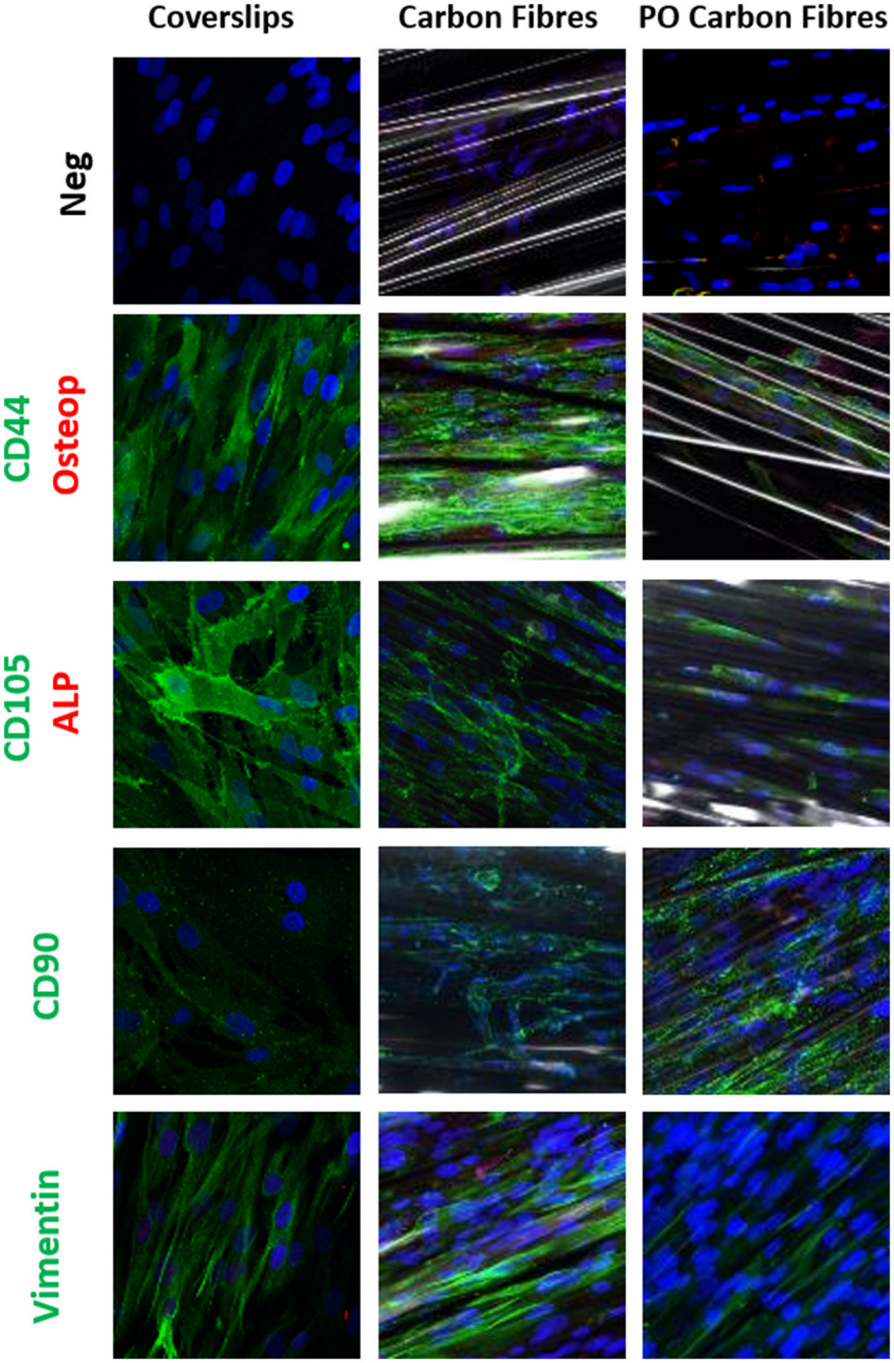
Single or dual immune staining of classic markers of the hADSCs on coverslips (control), the carbon fibers, and the plasma oxidized carbon fibers cultured over 3 weeks, where blue is nuclei stained with 4,6-diamidino-2-phenylindole (DAPI) immunostaining. The top row is a secondary negative control, row 2 shows positive green staining on CD44 and negative red staining of osteopontin, row 3 stains positive for CD105 marker (green) and negative staining for alkaline phosphatase (ALP) (red), row 4 stains positive for CD90 marker (green), and row 5 stains positive for vimentin marker (green).

**Figure 4:**
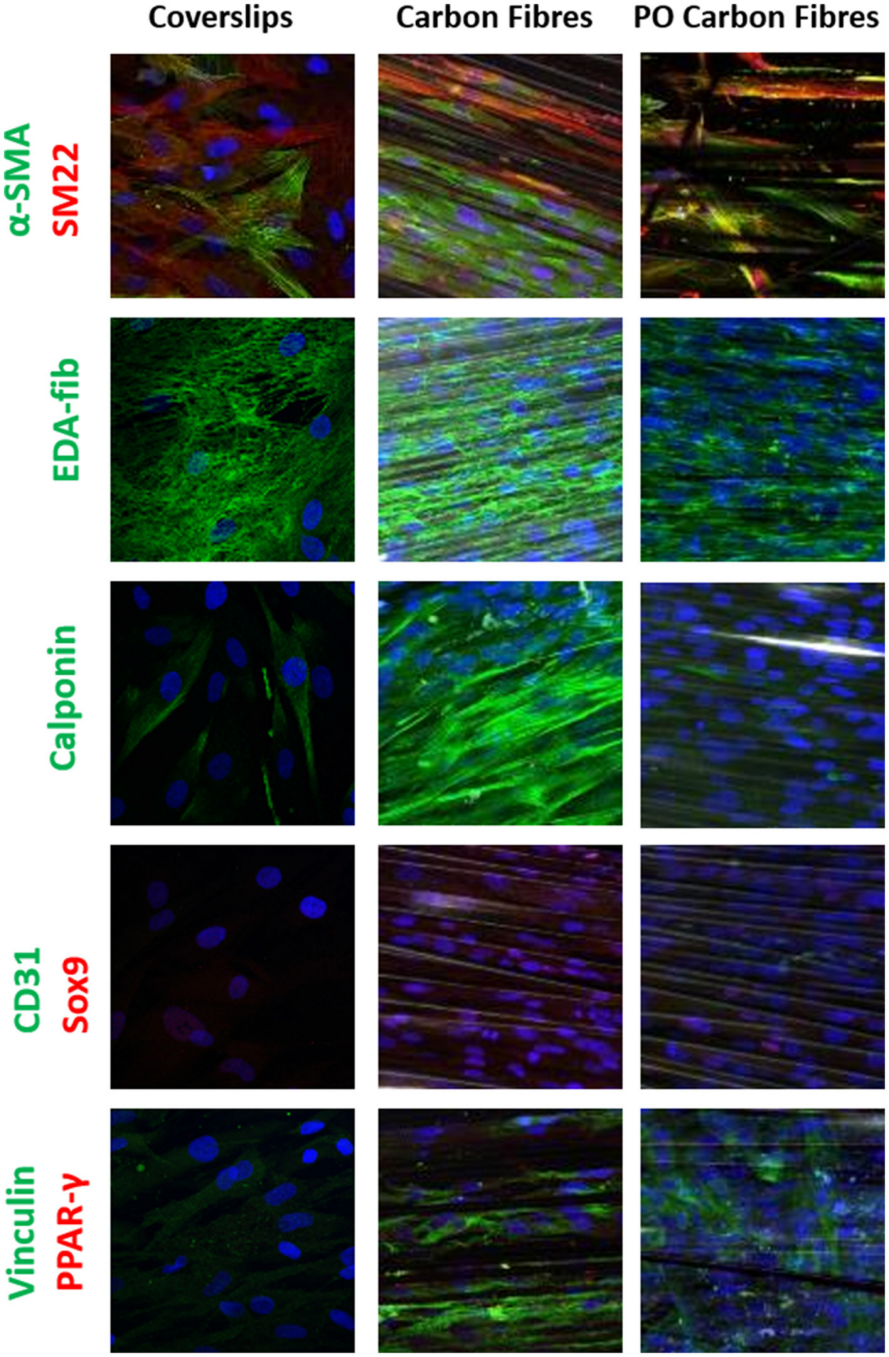
Single or dual immune staining of classic markers of the hADSCs on coverslips (as control), the carbon fibers, and the plasma oxidized carbon fibers cultured over 3 weeks, where blue is stained with DAPI immunostaining. The top row shows positive α-SMA (green) and SM22 (red) staining, row 2 shows positive EDA-fibronectin staining (green), row 3 shows positive calponin (green) staining, row 4 shows negative for CD31 (green) and Sox9 (red) staining, and row 5 shows positive vinculin (green) and negative for PPAR-γ (red).

Immunostaining was used to compare the phenotype of the hADSCs grown on coverslips, the pristine carbon fibers, and the plasma oxidized carbon fibers (Figures 3, 4). CD44 and CD105 were highly expressed on the hADSCs in all the 3 formats. CD90, another marker of mesenchymal stem cells, showed strong expression on both forms of the carbon fibers. The intermediate filament protein vimentin showed consistent staining of the hADSCs on all the formats.

Differentiation of the hADSCs was assessed by using markers for myofibroblastic, adipogenic, chondrogenic, and osteogenic differentiation. The hADSCs on coverslips showed weak homogeneous expression of SM22 (<20%) and a very low incidence of α-SMA (<10%) -positive hADSCs showed stress fiber staining. This expression was slightly higher between coverslips and the carbon fibers (Figure 4), indicating a low level of myofibroblastic activation. EDA-fibronectin, an early marker of myofibroblastic differentiation, showed enhanced expression on the untreated carbon fibers, but a similar low expression on the oxidized carbon fibers. Calponin showed a marked increase in expression on the untreated carbon fibers. There was no expression of CD31 on the hADSCs in any format; however, Sox9 showed weak expression in the hADSCs on the carbon fibers. There was no expression of PPARγ, osteopontin, or alkaline phosphatase. The expression of vinculin was enhanced on the carbon fibers (Figure 4).

### Mechanical Properties of the Carbon Fibers

The mechanical testing of the carbon fibers was performed with multifiber strands to mimic the application scenario. Stress/strain curves were generated (Supplementary Figure 2) using the carbon fibers in the longitudinal direction with the mechanical properties shown in Table 1. The stress-strain curve showed an initial toe region, which might have resulted due to the initial straightening of the multiple carbon fibers strands. The modulus of elasticity, ultimate tensile stress, and failure strain of the carbon fibers were 140 (±4.14), 3.52 (±0.11), and 0.039 Gpa (±0.0036), respectively.

**Table 1:** Mechanical properties of the carbon fibers measured and human heart valve from literature.

**Mechanical parameter**	**Carbon fiber**	**Heart valve (21)**
Modulus of elasticity	140 GPa (±4.14)	0.015 Gpa (circumferential) and 0.002GPa (radial)
Failure strain	0.039 (±0.0036)	0.22 (circumferential) and 0.3 (radial)
Ultimate tensile stress	3.52 GPa (±0.11)	0.0026 GPa (circumferential) and 0.0004GPa (radial)

### Analysis of the Range of Movement of Cusps and Sinuses

**Figure 5:**
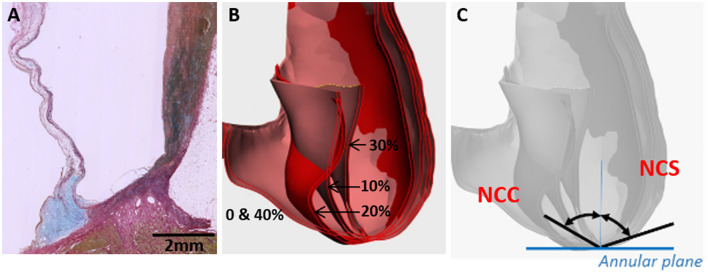
Cross-section through a normal human valvular root stained with Alcian blue (blue) and Sirius red (pink) showing the expression of glycosaminoglycans (blue) and collagens (pink), respectively **(A)**. The cusp is on the left and the sinus is on the right side. Overlaid CT images through a cross-section of a normal human valve at different phases of the systolic cycle (between 0 and 40% of the cardiac cycle is shown) **(B)** and angles, which were measured for all the 3 cusps and sinuses [non-coronary cusp shown in **(C)**].

**Figure 6:**
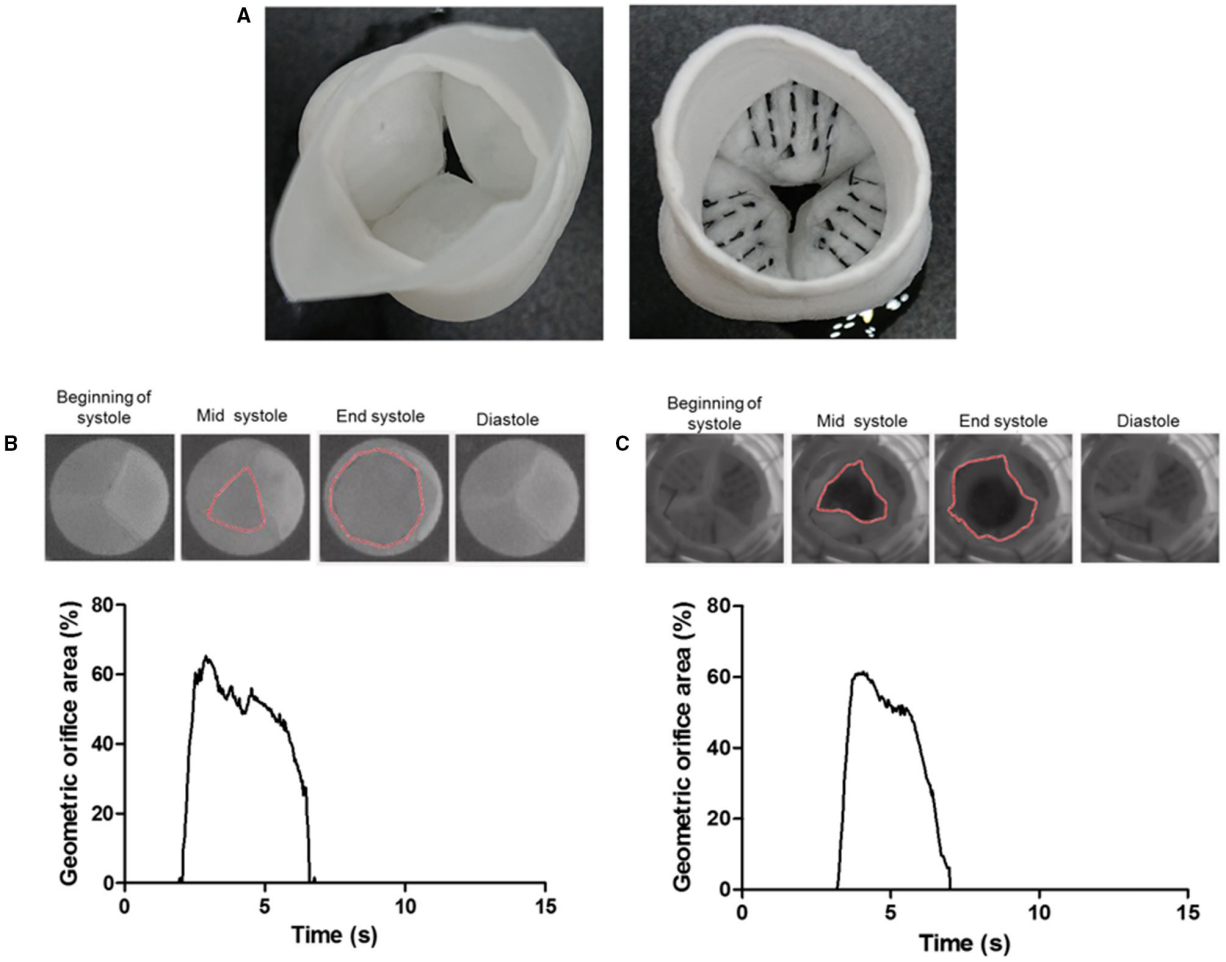
The ventricular view of a tissue-engineered valve root without the carbon fiber (left) and a prototype of a tissue-engineered valve root incorporated with the carbon fiber (right) along the hinge area **(A)**. Sample images of the opening and closure of a control valve root **(B)** and the carbon fiber embedded valve root **(C)** through a cardiac cycle in a pulse duplicator. The corresponding graph shows the tracking of their geometric orifice area (GOA) through a cardiac cycle. Both types of valves show a similar maximum GOA at around 60%.

An example of a normal human valvular root stained with Alcian blue is shown in Figure 5A. The cusp of the valve is hinged onto the sinus wall as part of its structural support. Changes in the angles of each cusp and sinus over the cardiac cycle were measured in the region as shown in Figures 5B, 6C. It showed that the cusps—non-coronary cusp (NCC), right coronary cusp (RCC), and left coronary cusp (LCC) had a greater range of motion of 9–70° compared to a limited range of motion, 30–48°, for the coronary sinuses (Table 2).

**Table 2:** The angle of the cusps and sinuses formed to the perpendicular line going through the nadir of the annulus at different phases of the cardiac cycles.

**Phase**	**NCC**	**NCS**	**RCC**	**RCS**	**LCC**	**LCS**
0%	70°	33°	62°	41°	68°	36°
10%	37°	38°	14°	30°	29°	34°
20%	44°	42°	9°	32°	20°	42°
30%	27°	48°	11°	41°	23°	44°
40%	70°	33°	62°	41°	68°	36°
Range	27–70°	33–48°	9–62°	30–41°	20–68°	34–44°

*NCC, non-coronary cusp; NCS, non-coronary sinus; RCC, right coronary cusp; RCS, right coronary sinus; LCC, left coronary cusp; LCS, left coronary sinus*.

Measurements of the radii of the sinuses and cusps during valve opening and closure, and, consequently, of the curvature, showed a maximum range of 0.09–0.50 for the LCC and 0.08–0.15 for the corresponding sinus, left coronary sinus (LCS) (Table 3). Curvature was similarly greater for the NCC and RCC compared to their corresponding sinuses.

**Table 3:** Radius (mm) of the first third of cusp mid-curve and sinus mid-curve.

**Phase**	**NCC**	**NCS**	**RCC**	**RCS**	**LCC**	**LCS**
0%	12	5	11	12	11	6.5
10%	3	6	3	9	2	12
20%	4.5	5.5	4	10	5	8
30%	3	7	2.5	11	2	11
40%	12	5	11	12	11	6.5
Range	3–12	5–7	2.5–11	9–12	2–11	6.5–12

*NCC, non-coronary cusp; NCS, non-coronary sinus; RCC, right coronary cusp; RCS, right coronary sinus; LCC, left coronary cusp; LCS, left coronary sinus*.

### Carbon Fiber Reinforced Cusp and Geometric Orifice Area of the Valve

To demonstrate the proof-of-principle that the carbon fibers can be embedded into PCL-sprayed nanofibers and maintained normal valvular cusp function, functional PCL nanofibrous heart valve roots with and without the carbon fibers (Figure 6A) were subjected to hydrodynamic testing in a pulse duplicator. The geometric orifice area at the end of the systolic phase in the model without the carbon fibers was 65% and this was very similar to the model with the carbon fibers at 62%. Both the models closed fully in the diastolic phase (Figures 6B,C).

## Discussion

In a load-bearing application such as the heart valve, biodegradable materials present a significant challenge in balancing the rate of polymer degradation vs. the continued mechanical function of the construct (2). Therefore, a strategy that incorporates the carbon fibers into the tissue-engineered constructs to ensure the continued function is proposed in this study.

Carbon fiber is a well-established material that is currently used in the construction industry such as suspension bridges for its superior strength, fatigue resistance, durability, flexibility, and elastic recovery. Thus, strategic incorporation of the carbon fibers into a biodegradable scaffold can ensure the continued load-bearing function of the targeted tissue. In addition, previous *in-vitro* and *in-vivo* studies on the other carbon fibers have yielded controversial results showing that the carbon fibers induced the growth of new tissue (11, 22) and other studies yielded opposite results (23, 24). Bone, ligaments, and tendon application have been previously the main focus of biocompatibility studies for carbon fibers. With the increased interest in regenerative medicine and tissue engineering, the interaction of the carbon fibers with stem cells is now relevant but has not been tested. In this study, we demonstrate that the carbon fibers are compatible with the hADSCs, support ECM deposition as evidenced by the expression of EDA-fibronectin, have superior strength, and are flexible enough to allow the free movement of the valve cusps when stitched into a tissue-engineered valve construct from the sinus wall, across the hinge region and into the belly of the cusp. This study has shown the potential use of these carbon fibers in heart valve tissue engineering.

We chose to examine the biocompatibility of the carbon fibers with the hADSCs since these cells are good candidates in seeding scaffolds for *in-vitro* tissue engineering strategies (25). In addition, the differentiation capacity of the hADSCs permits these cells to serve as an indicator for conditions that may favor the expression of adipogenic, chondrogenic, and osteogenic cell phenotypes (26). The hADSCs were able to adhere to the smooth surface of the pristine carbon fibers as shown with the SEM images and the MTS assay (Figure 2). Furthermore, the number of cells adhering could be significantly enhanced by prior activation of the surface by plasma oxidation, a process that leads to the production of acid oxides on the surface of the carbon fibers that enhances surface hydrophilicity, thereby enhancing surface activation energy suitable for matrix bonding (27).

The hADSCs that were cultured onto the carbon fibers retained their stem cell phenotype with no evidence of differentiation into adipogenic, chondrogenic, osteogenic, or endothelial cell phenotypes, However, there was some myofibroblastic differentiation with upregulation of α-SMA, calponin, and EDA-fibronectin. The plasma oxidized carbon fiber reduced this level of activation. The lack of differentiation to other phenotypes indicates that the cells are essentially inert to the carbon fibers as previously reported (28). With respect to *in vitro* heart valve tissue engineering, the use of hADSCs and the carbon fibers may prove useful especially, as the hADSCs were shown to retain their phenotype and specific differentiation can be induced and guided by the application of growth factors, peptides, and compounds. We have previously shown that using an active lysine-threonine-threonine-lysine-serine (KTTKS) peptide motif enhanced the secretion of ECM components (29) and using specific motifs can drive the expression of tissue-specific ECM proteins. Combining surface activation with plasma oxidation, the carbon fibers can be easily linked to specific bioactive peptides or biomolecules through carbodiimide chemistry.

The native heart valves have mechanical stiffness in the range of 1 to 2 MPa in the radial and 10 to 20 MPa in the circumferential directions, with ultimate tensile stress (UTS) of 0.4 MPa in the radial and 2.6 MPa in the circumferential directions (21, 30, 31) as shown in Table 1 and a typical polymeric porous scaffold used in heart valve tissue engineering has significantly lower mechanical stiffness in the range of 3 to 6 MPa and UTS in the range of 0.4 to 0.7 MPa (31) due to its porous nature to allow for cell colonization. Furthermore, tissue-engineered scaffolds suffer from further deterioration during long implantation periods due to biodegradation and repetitive stress. The engineering application of carbon fibers has been used extensively as a reinforcement component in composite materials due to their ultra-high strength. Therefore, the carbon fiber could be used to form part of a composite scaffold to reinforce it. Mechanical testing of the carbon fibers showed them to have an extremely high modulus of 140 GP (±4.14) and ultimate tensile strength at 3.52 Gpa (±0.11) in the direction of the carbon fibers. These fall in the range of the other carbon fibers produced from PAN and mesophase pitch (MPP) (32).

Despite the high modulus of the carbon fibers, one important design constraint with the carbon fibers was that they became brittle and snapped if they were forced to bend at a sharp 90° angle. This has been reported previously when used in reconstruction for chronic anterior cruciate ligaments, where they found that the carbon fibers broke under twisting or angular forces (33). The brittleness of the carbon fibers at a sharp 90° angle could be a design constraint for heart valve tissue engineering. As a first step, we calculated the angle between the sinus wall and the valve cusp varied during the opening and closing phases of the valve. CT-based analysis of the movement of the aortic cusps in relation to each corresponding sinus showed a great range of movement of the cusps and a maintained curvature at the hinge area, despite the significant changes in angles in the hinge area. These calculations showed that the angle between the sinus wall and each of the three valve cusps did not exceed a 90° angle. Furthermore, as a proof-of-principle, the embedding of carbon fibers across the radial direction of the tissue-engineered heart valve showed that the geometric orifice area and leaflet motion of a tissue-engineered valve in a bioreactor were unaffected by the incorporation of the carbon fibers. In this configuration, the carbon fibers utilized the sinus wall as a pillar in a suspension bridge to transfer the load on the valvular cusp during the diastolic phase, while allowing the heart valve to open without significant obstruction.

This study establishes the potential utility of the carbon fibers in tissue-engineered heart valves. There remain several additional studies that are required to assess if the carbon fibers will provide any benefit to the durability and function of tissue-engineered heart valves. The carbon fibers used in this study were sewn into the cusps in a radially orientated line across the width of the cusp; these carbon fibers may not necessarily be the optimal width apart or in the best orientation. Further studies are required to establish the potential long-term benefits of the reinforcement of scaffold material on the durability and functions of tissue-engineered heart valves both *in vitro* and *in vivo* studies. This study has used one cell type to assess the biocompatibility of the carbon fibers. Previous studies have also shown the carbon fibers to be compatible with cells, but this may be dependent upon the types of carbon fiber used (34–36). Assessment of the cellularization of scaffold materials containing the carbon fibers *in vivo* will be the ultimate test of biocompatibility.

## Conclusion and Future Study

In this study, we demonstrated that the carbon fibers can be populated by the hADSCs without stimulating their differentiation. The carbon fibers were sufficiently flexible to be incorporated into an *in vitro* functioning tissue-engineered heart valve without restricting the motion of the cusps. Further study is required to optimize the carbon fiber distribution/pattern and embedding method in order to optimize their potential to enhance the durability and hemodynamic performance of tissue-engineered heart valves.

## Data Availability Statement

The original contributions presented in the study are included in the article/Supplementary Material, further inquiries can be directed to the corresponding author/s.

## Ethics Statement

The studies involving human participants were reviewed and approved by Aswan Heart Science Centre Ethics Committee. The patients/participants provided their written informed consent to participate in this study.

## Author Contributions

YT-T and NL contributed to the experimental plan, concept, data collection, analysis, and writing of the manuscript. NG and HA contributed to the concept. PS, AM, AE, NS, MN, and H-EN contributed to the data collection and analysis. MY and AC involved in the concept and editing and reviewing of the manuscript. All authors contributed to the article and approved the submitted version.

## Funding

We would like to thank the Magdi Yacoub Institute for funding this study.

## Conflict of Interest

The authors declare that the research was conducted in the absence of any commercial or financial relationships that could be construed as a potential conflict of interest.

## Publisher's Note

All claims expressed in this article are solely those of the authors and do not necessarily represent those of their affiliated organizations, or those of the publisher, the editors and the reviewers. Any product that may be evaluated in this article, or claim that may be made by its manufacturer, is not guaranteed or endorsed by the publisher.
