# Supplementary material for: Biocompatibility and Application of Carbon Fibers in Heart Valve Tissue Engineering
Source: Front Cardiovasc Med. 2021 Dec 24;8:793898. doi: 10.3389/fcvm.2021.793898 (PMC8739227; doi:10.3389/fcvm.2021.793898)
Supplement: Supplementary file 1 [file Data_Sheet_1.docx]

# Supplementary information

# Method

## Flow Cytometry

hADSCs (10^5^ cells) were resuspended in 100 μL of PBS and stained with primary antibodies for one hour. Monoclonal antibodies against CD44 (Serotec), CD73 (BD), CD105 (BD), CD45 (Abcam), CD31 (DAKO) and CD68 (DAKO) were used. Excess primary antibody was removed in two washes before addition of FITC goat antimouse IgG (Dako) for one hour. Unbound antibody was removed in two washes, and cells were fixed in 400 μL of 0.05% formaldehyde (BDH, Essex, UK). Ten thousand events were acquired on an EPICS XL flow cytometer (Beckman Coulter). The negative controls (with no primary antibody staining or stained with isotype control) always provided less than 2% of total cell staining. This background figure was subtracted from the percentage of cells staining positively with monoclonal antibodies.

## Differentiation of hADSCs

Cultured cells at passage 3 were cultured in adipogenic differentiation medium (DMEM-LG containing 10% FCS, 50 mM L-ascorbic acid-2-phosphate, 10–7 M dexamethasone and 50 µg/ml indomethacin (Sigma, UK); in chondrogenic differentiation medium (as cell pellets) (serum-free DMEM-LG containing insulin transferrin-selenium (ITS) + premix (Invitrogen, UK) and 10 ng/ml transforming growth factor (TGF)-β1 (Pepro Tech, Rocky Hill, NJ, USA); osteogenic differentiation medium (DMEM-LG containing 10% FCS, 100 nM dexamethasone, 10 mM α-glycerophosphate and 50 mM L-ascorbic acid-2- phosphate); or in DMEM-LG supplemented with 10% FCS as a control. The medium was changed every three days, and differentiation was established by morphology and histochemical staining. Adipogenic differentiation was demonstrated by Oil red O staining, chondrogenic lineage formation was demonstrated by Safranin-O staining and osteogenic differentiation was demonstrated by alkaline phosphatase staining.

# Results

## Flow Cytometry

Expanded hADSCs were phenotyped using FACS for classical mesenchymal stem cell markers CD44, CD73 and CD105 as well as exclusion markers CD45, CD31, CD68 to confirm stem cell maintenance pre-seeding on carbon fibres. The negative control was set to 2.0%. The classical stem cell markers, CD44, CD73, CD90 and CD105 were all highly (>98.0%) expressed with no expression of exclusion markers CD31 (1.4% ± 0.6) or CD45 (1.2% ± 0.8), (Suppl Fig. 1), Table 1.

## Supplementary Figure 1.


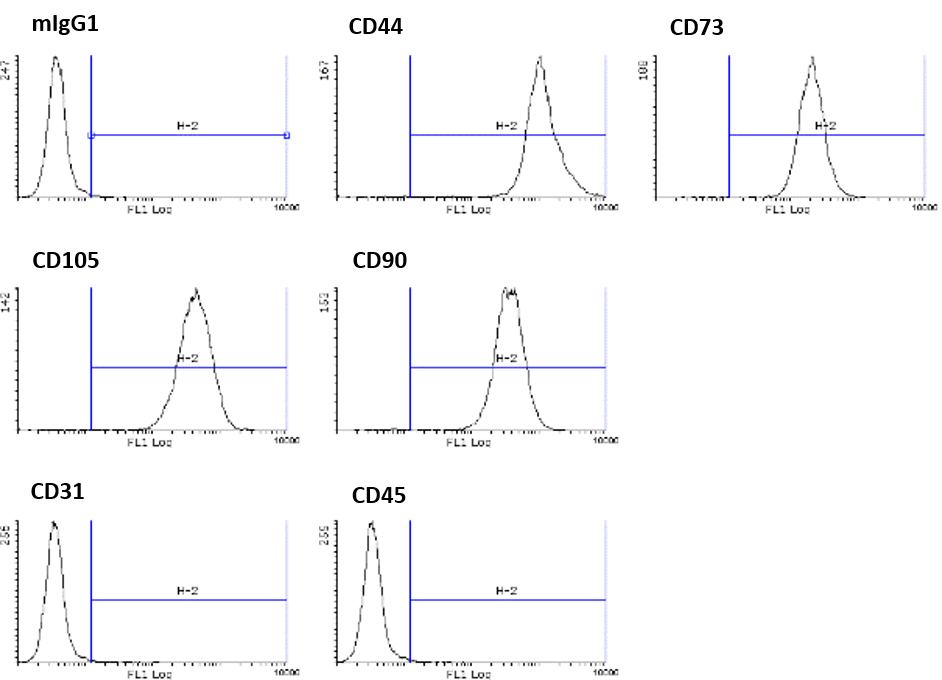


FACS histograms showing the phenotype of the hADSCs with the negative control (no antibody**,** isotype control)**,** positive markers, CD44, CD73, CD105 and CD90 and negative markers CD31 and CD45.

## Supplementary Table 1.

Table 1.

| Antigen | % Fluorescence | MFI |
| --- | --- | --- |
| -ve | 1.0 ± 0.4 | 5 ± 0.3 |
| CD44 | 98.8 ± 2.3 | 1370 ± 21.3 |
| CD73 | 99.6 ± 2.1 | 227 ± 1.7 |
| CD90 | 98.9 ± 3.4 | 412 ± 0.9 |
| CD105 | 98.9 ± 4.2 | 496 ± 2.2 |
| CD31 | 1.4 ± 0.6 | 9 ± 1.2 |
| CD45 | 1.2 ± 0.8 | 8 ± 1.6 |

## Supplementary Figure 2.


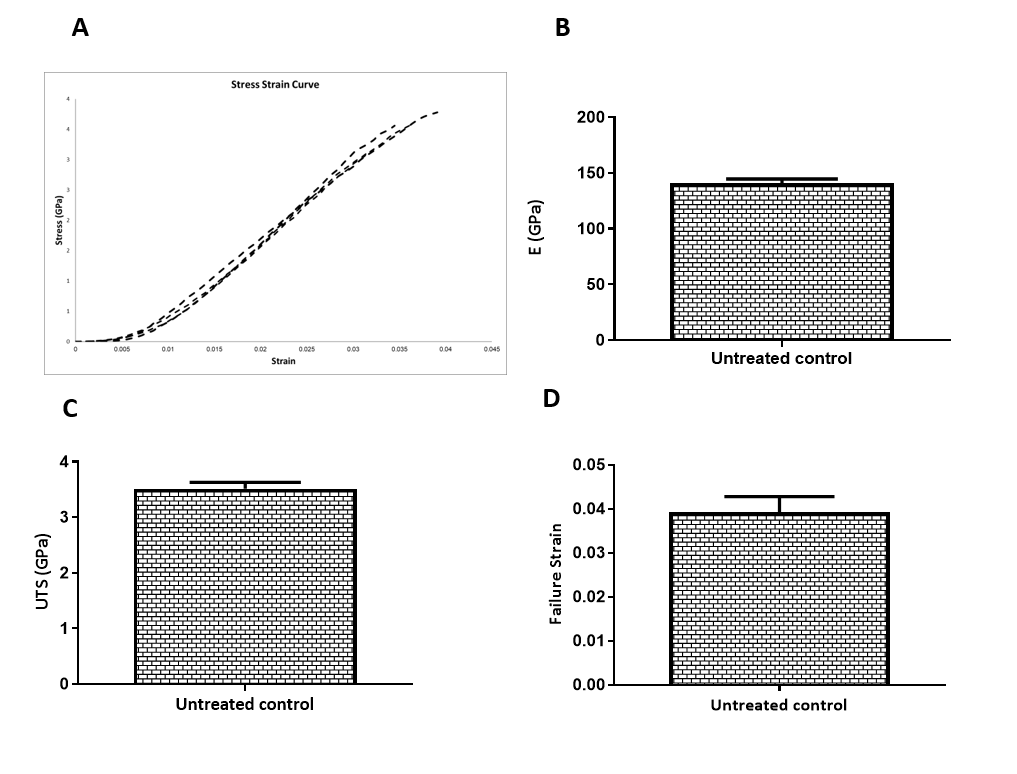


1. typical stress strain curves of carbon fibres B) Young’s modulus carbon fibres, C) ultimate tensile strength of carbon fibres, D) failure strain of carbon fibres. The typical stress-strain of carbon fibers shows initial toe region due to fiber strengthening, followed by linear increase in stress and strain relationship.

## Supplementary Figure 3.


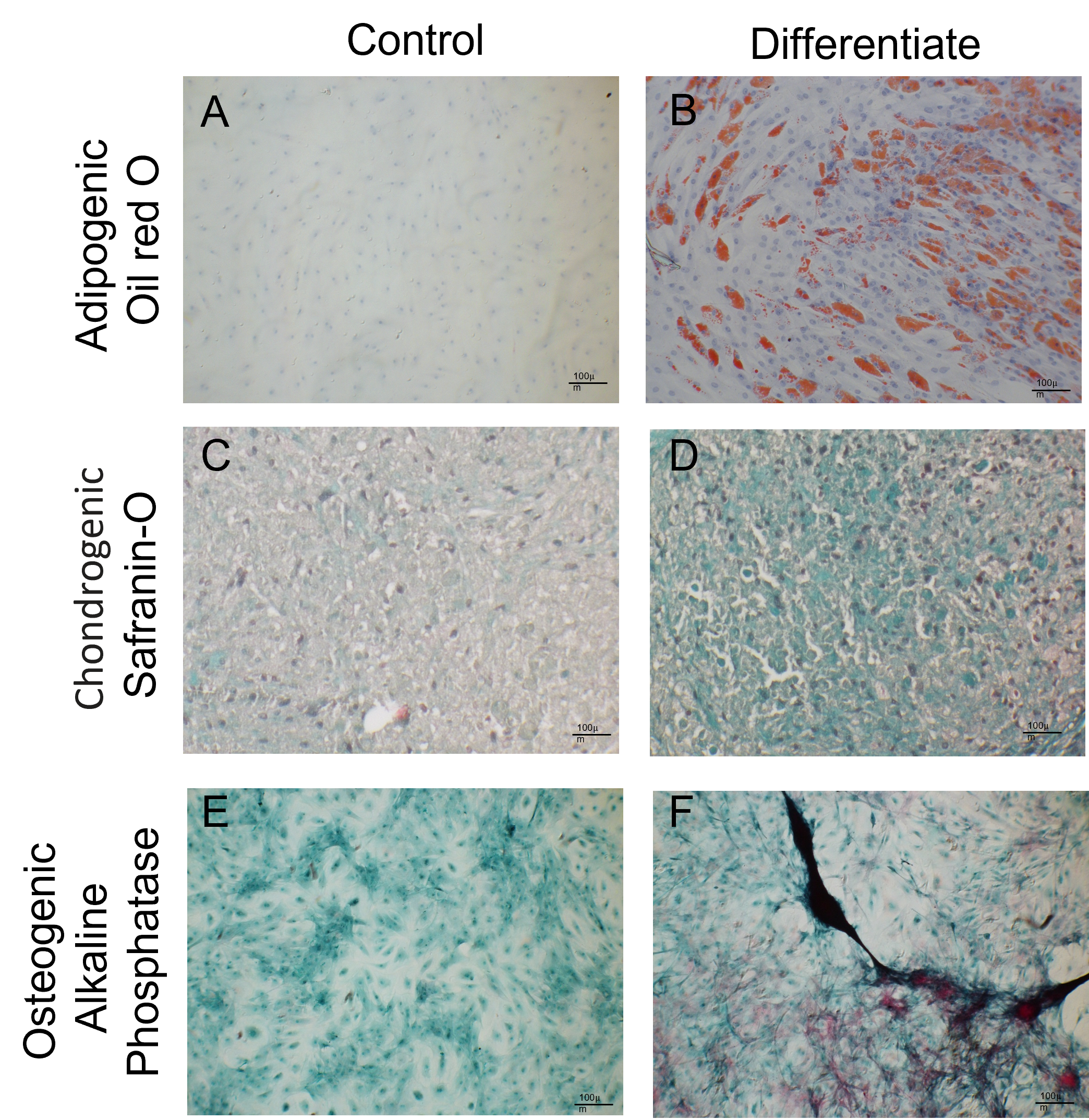


Histochemical staining of 3 weeks differentiation induction of hADSCs at 10× magnification. A and B are negative control and differentiated with adipogenic protocol respectively, stained with oil red O. C and D are negative control and differentiated with chondrogenic protocol respectively, with Safranin-O staining. E and F are control and differentiated with osteogenic protocol with alkaline phosphatase staining.   Scale bars are 100µm.
